# Allergic Rhinitis: A Clinical and Pathophysiological Overview

**DOI:** 10.3389/fmed.2022.874114

**Published:** 2022-04-07

**Authors:** Siti Muhamad Nur Husna, Hern-Tze Tina Tan, Norasnieda Md Shukri, Noor Suryani Mohd Ashari, Kah Keng Wong

**Affiliations:** ^1^Department of Immunology, School of Medical Sciences, Universiti Sains Malaysia, Kubang Kerian, Malaysia; ^2^Department of Otorhinolaryngology, Head and Neck Surgery, School of Medical Sciences, Universiti Sains Malaysia, Kubang Kerian, Malaysia; ^3^Hospital Universiti Sains Malaysia, Kubang Kerian, Malaysia

**Keywords:** allergic rhinitis, epidemiology, diagnostic criteria, pathophysiology, Th2 responses, immunotherapy

## Abstract

Allergic rhinitis (AR) represents a global health concern where it affects approximately 400 million people worldwide. The prevalence of AR has increased over the years along with increased urbanization and environmental pollutants thought to be some of the leading causes of the disease. Understanding the pathophysiology of AR is crucial in the development of novel therapies to treat this incurable disease that often comorbids with other airway diseases. Hence in this mini review, we summarize the well-established yet vital aspects of AR. These include the epidemiology, clinical and laboratory diagnostic criteria, AR in pediatrics, pathophysiology of AR, Th2 responses in the disease, as well as pharmacological and immunomodulating therapies for AR patients.

## Introduction

Atopic allergic sensitization is defined by the production of immunoglobulin E (IgE)-mediated immune response toward allergens. Allergic rhinitis (AR) is a common disorder that afflicts 400 million people worldwide and it represents a global concern as its prevalence has increased over the years (1). AR usually comorbids with other diseases such as asthma (2), leading to impaired quality of life, school or work performance, and significant financial impact. AR is shown to be caused by aberrantly high Th2 cytokines, and recent findings on the cause of AR are directed toward impairment of the nasal epithelial barrier integrity (3–8). In this review, we summarize the well-established yet important concepts of AR including the epidemiology, clinical and laboratory diagnostic criteria, pathophysiology of AR including allergens as well as Th2 responses in AR.

## Epidemiology of Allergic Rhinitis

Allergic rhinitis prevalence has increased significantly since the 1990s (9–11). It is reported to affect approximately 25 and 40% of children and adult globally, respectively. Approximately 80% of AR symptoms develop before the age of 20 years (12) and peak at age 20–40 years before gradually declining (13). The incidence rate of AR in children over the first 5 years of life was reported to be 17.2%, with a peak age at diagnosis between 24 and 29 months (2.5%) (14). Meta-analysis studies have shown the sex-specific differences in the prevalence of AR with male predominance in childhood and a female predominance in adolescents (15, 16).

Prevalence of AR has increased with years due to several risk factors including global urbanization as shown by several studies comparing AR prevalence in urban settings with rural areas (17, 18). This is mainly caused by increased levels of pollutants [e.g., traffic-related pollutants and particulate matter 2.5 (PM2.5)] that can exacerbate pollen-sensitized AR (19–21). It has been reported that AR is more prevalent in urban areas compared with rural areas (18). Climate changes also prolong pollen season as reported in Europe over the last three decades along with more frequent seasonal allergies (22).

Smoking, however, did not show a significant association with the severity of nasal symptoms in AR but usually impacted patients with chronic rhinitis (23, 24). Conversely, maternal smoking conferred the greatest risk in pediatric AR (25). Novel tobacco products such as electronic cigarette and heated tobacco products increase the risk of AR in adolescents compared with traditional smoking (26).

The economic impact of AR is significant where the total annual cost of self-reported AR in Sweden is estimated at €1.3 billion (27) and up to $20.9 billion in United States (1). In addition, AR is a systemic inflammatory disease and often comorbids with other disease such as asthma, atopic disease, sinusitis, conjunctivitis, and otitis media (9), complicating the treatment and management of AR patients.

## Clinical Signs and Symptoms of Allergic Rhinitis

AR is characterized by the presence of nasal and non-nasal symptoms. Nasal symptoms include anterior or posterior rhinorrhea, sneezing, nasal blockage and/or itching of the nose (9). These symptoms may persist for hours after allergic reaction upon the exposure of allergens that cause mucosal inflammation (13). In consequences, the mucosa is rendered more reactive to the triggering allergen as well as to other allergens and to non-allergenic stimuli (e.g., strong odors and other irritants). Non-nasal symptoms are characterized by ocular symptoms such as allergic rhinoconjunctivitis (i.e., itching and redness of the eyes and tearing) which frequently occurs in AR patients (10). Other symptoms include itching of the palate, postnasal drip and cough.

Hypersensitivity reactions can be observed in AR, bronchial asthma, allergic conjunctivitis, allergic dermatitis, food allergy and anaphylactic shock (28). Over 30% of AR patients suffer debilitating allergic symptoms that can lead to severe disability and life-threatening conditions such as anaphylaxis (29). In severe cases, intense bronchospasm, laryngeal edema, cyanosis, hypotension and shock may occur (28).

In terms of AR severity, it can be classified as mild and moderate/severe based on the AR and its Impact on Asthma (ARIA) guidelines (9). It is measured based on four aspects including sleep abnormality, impairment in daily activities, impairment in school or work performance, and troublesome symptoms. Patients without the aforementioned problems are considered as mild AR while patients suffering from one or more of the items are considered as moderate/severe AR. The ARIA guidelines also classify AR symptoms into intermittent and persistent based on the duration of symptoms present in AR patient. For intermittent symptoms, the symptoms occur in less than 4 days per week or less than 4 consecutive weeks while for persistent symptoms, they occur in more than 4 days per week and more than 4 consecutive weeks.

## Laboratory Characteristics of Allergic Rhinitis

To determine the specific allergen that causes the production of IgE antibodies in AR, multiple tests can be conducted such as *in vivo* skin tests including skin prick test (SPT; percutaneous) and intradermal (intracutaneous) skin tests (IDST), and *in vitro* serum allergen-specific IgE (ssIgE) immunoassay. SPT and ssIgE immunoassay are the most common laboratory tests to determine the causative allergen(s) (30, 31). However, there is no “gold standard” laboratory test in diagnosing AR but SPT represents the first-line approach in the assessment of allergic sensitivities (9, 32). SPT is a quick and cost-effective methodology in diagnosing any allergic sensitization (33).

ssIgE immunoassay utilizes commercially available test panels which are more costly (33). It is also less sensitive for the diagnosis of allergy due to inhalant allergens compared with SPT. However, ssIgE immunoassay can be useful when skin testing is not available or cannot be performed due to patients have extensive skin disease, unable to discontinue antihistamines or other interfering medications, dermatographic, or other issues that complicate skin testing (34, 35). A novel non-invasive local diagnosis is through dried blood spot (DBS)-based diagnosis to detect IgE reactivity which can test up to more than 170 allergen molecules (36, 37).

Another uncommon diagnostic test for AR includes intradermal skin testing (IDST) in which tiny quantity of allergen is injected into the dermis with a hypodermic needle for the diagnosis of IgE-mediated allergic conditions (38). Other alternative or supporting tests include eosinophil cationic protein (ECP) and the percentage of eosinophils (39, 40), tryptase (marker of mast cell activation) (41), leukotriene B4 (40) and basophil activation test (through flow cytometry) to determine the causative allergen in local AR (42, 43). These tests are usually conducted in research settings and not routinely used for AR diagnosis.

## Diagnostic Criteria of Allergic Rhinitis

Diagnostic criteria are generally broad and must reflect the different features of a disease (i.e., heterogeneity), with a view to accurately identify as many patients with the condition as possible (44). Due to the lack of gold standards in diagnosing AR, definitive diagnostic criteria have been challenging to establish. The choice of confirmatory test is a matter of clinical judgment and the results obtained must be considered together with additional risk factors, rather than definitive indicators of disease (45).

However, for patients to be diagnosed with AR, they must have clinical symptoms and possess laboratory characteristics as discussed in the previous section. AR patients must encounter two or more of the following clinical symptoms for more than 1 h on most days: (1) Watery rhinorrhea; (2) Sneezing, especially paroxysmal; (3) Nasal obstruction; (4) Nasal pruritis; (5) With or without conjunctivitis. When individuals present with clinical symptoms of AR, allergy laboratory tests are conducted for confirmation.

**FIGURE 1:**
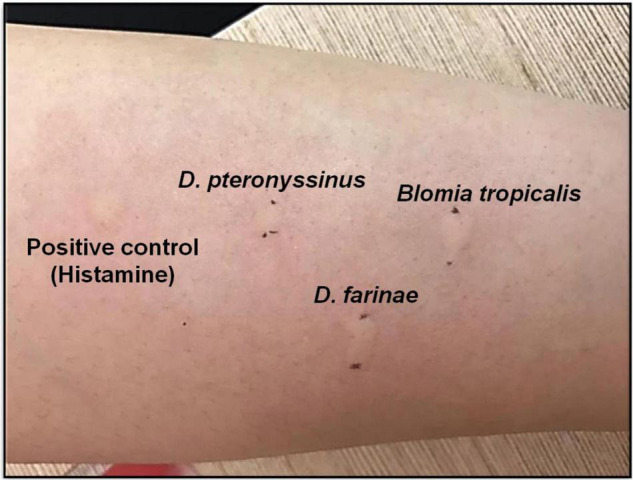
Positive SPT of HDM allergen *Dermatophagoides pteronyssinus* (*D. pteronyssinus;* wheal size 9 mm), *Dermatophagoides farinae* (*D. farinae*; wheal size 11 mm) and *Blomia tropicalis* (*B. tropicalis*; wheal size 10 mm) tested on an AR patient at ORL-HNS clinic, Hospital Universiti Sains Malaysia.

In skin tests, positive result is considered when the wheal-and-flare reaction occurs on the skin test site after 20 min of exposure to allergens. For SPT, positive result must demonstrate wheal (i.e., a red and itchy raised bump with surrounding inflammation that indicates the presence of allergic antibodies) size in diameter of ≥4 mm (46, 47). Figure 1 shows a positive SPT on house dust mite (HDM) allergens (*Dermatophagoides pteronyssinus, Dermatophagoides farinae* and *Blomia tropicalis*) tested on an AR patient who attended the ORL-HNS clinic of Hospital Universiti Sains Malaysia, and each wheal size was greater than 4 mm. The SPT protocols were approved by the Human Research Ethics Committee of Universiti Sains Malaysia (JEPeM; approved ethics code: USM/JEPeM/18060273).

The performance of any diagnostic criteria is dependent on the prevalence of the disease in a given geographical area or clinical setting (e.g., community clinic vs. tertiary care facility). AR diagnostic criteria are summarized in Table 1.

**TABLE 1:** Diagnosis criteria of allergic rhinitis (AR) based on clinical symptoms and laboratory characteristics criteria (9).

Clinical symptoms criteria	Laboratory characteristics criteria
Two or more of the following symptoms for >1 h on most days:	At least one of the laboratory characteristics:
• Watery rhinorrhea	• Positive SPT (wheal diameter of ≥ 4 mm)
• Sneezing, especially paroxysmal	• Positive IDST (wheal diameter of ≥ 5 mm)
• Nasal obstruction	• Total serum IgE (general; > 100 kU/L, >14 years; > 333 kU/L)
• Nasal pruritus	• Serum allergen-specific IgE (>0.35 kU/L)
• With or without conjunctivitis	• Blood eosinophil count (>80 cells/ml/>5–10% of total WBC count)
	• Total serum tryptase level (>20 μg/L)

## Pediatric Allergic Rhinitis

Allergic rhinitis represents a common pediatric problem where approximately 40% of pediatric AR patients develop symptoms as early as age 6 years old and increase with age (48–50). Symptoms of pediatric AR are similar with adolescents; however, young children may frequently exhibit sniffing, snorting, throat-clearing, and coughing (51). Comorbidities in pediatric AR are often combination of several conditions which indicate AR is a disease manifestation that involves systemic inflammation including conjunctivitis, atopic dermatitis, asthma, rhinosinusitis, otitis media with effusion, or food allergies (51, 52). The MeDALL (Mechanisms of the Development of ALLergy) project that involved more than 12,000 children from 12 ongoing longitudinal cohort studies showed the co-existence of asthma, rhinitis and eczema prevalence in the same child (53). In addition, findings from the Copenhagen Prospective Studies on Asthma in Childhood 2010 (COPSAC_2010_) have shown higher prevalence of AR, asthma and aeroallergen sensitization in children exposed to urban environment in their early age compared with those exposed to rural environment (54).

Diagnosis of AR in pediatrics includes complete history taking, physical examinations (e.g., nose, oropharynx, tympanic membranes, and eyes) as well as differential diagnosis based on the clinical symptoms suggestive of AR but not evidence of systemic atopy (55). A very young child with persistent nasal symptoms should be considered as having other disorders that can mimic AR (56). For children under 2 years of age, other disorders include adenoidal hypertrophy, acute or chronic sinusitis, congenital abnormalities (choanal atresia), foreign bodies and nasal polyps will be taken into consideration during diagnosis. On the other hand, older children may present with other disorders including acute infectious rhinitis, chronic non-allergic rhinitis, chronic rhinosinusitis, rhinitis medicamentosa, rhinitis due to systemic medications, atrophic rhinitis, rhinitis associated with hormonal changes, unilateral rhinitis or nasal polyps and rhinitis with immunologic disorders. Descriptions on each of these specific disorders (56) and differential diagnosis based on age (57) have been discussed in the cited references.

## Pathophysiology of Allergic Rhinitis

### Early and Late Phase of Allergic Rhinitis

Type I hypersensitivity is an allergic reaction mediated by IgE antibody in response to allergens (13). Type I hypersensitivity reactions occur rapidly, usually within 20 min after allergen exposure, and it is characterized by activation of mast and inflammatory cells as well as their infiltration in tissues (58). The allergic response in AR can be divided into two phases i.e., the early and late phase.

The early phase starts within 20 min after exposure to harmful allergens. Antigen presenting cells such as dendritic cells in the mucosal surface uptake, process and present peptides from allergens on the major histocompatibility complex (MHC) class II molecule. The antigen complex and the MHC class II molecule serve as a ligand for T cell receptors on naïve CD4^+^ T cells, resulting in differentiation of naïve CD4^+^ T cells into allergen-specific Th2 cell. Cytokines such as IL-4 and IL-13 released from the activated Th2 cells interact with B cells to produce allergen-specific IgE. This allergen-specific IgE binds to high-affinity Fc receptor for IgE (FcεR) present on mast cells, leading to mast cell activation (6, 59, 60).

Cross-linking of the FcεR on mast cells causes release of allergic mediators consisting of histamine, proteases and lipid mediators such as leukotriene (LT) C4, and prostaglandin D2 (PGD2) that cause vascular leak, bronchoconstriction, inflammation, and intestinal hypermotility (28, 61–63). These mediators induce mucosal edema and watery rhinorrhea characteristic of AR by causing the blood vessels to leak. Histamine is the major mediator in AR where it activates H1 receptors on sensory nerve endings and causes sneezing, pruritus, and reflex secretory responses, and it also interacts with H1 and H2 receptors on mucosal blood vessels, leading to vascular engorgement (nasal congestion) and plasma leakage (64).

**FIGURE 2:**
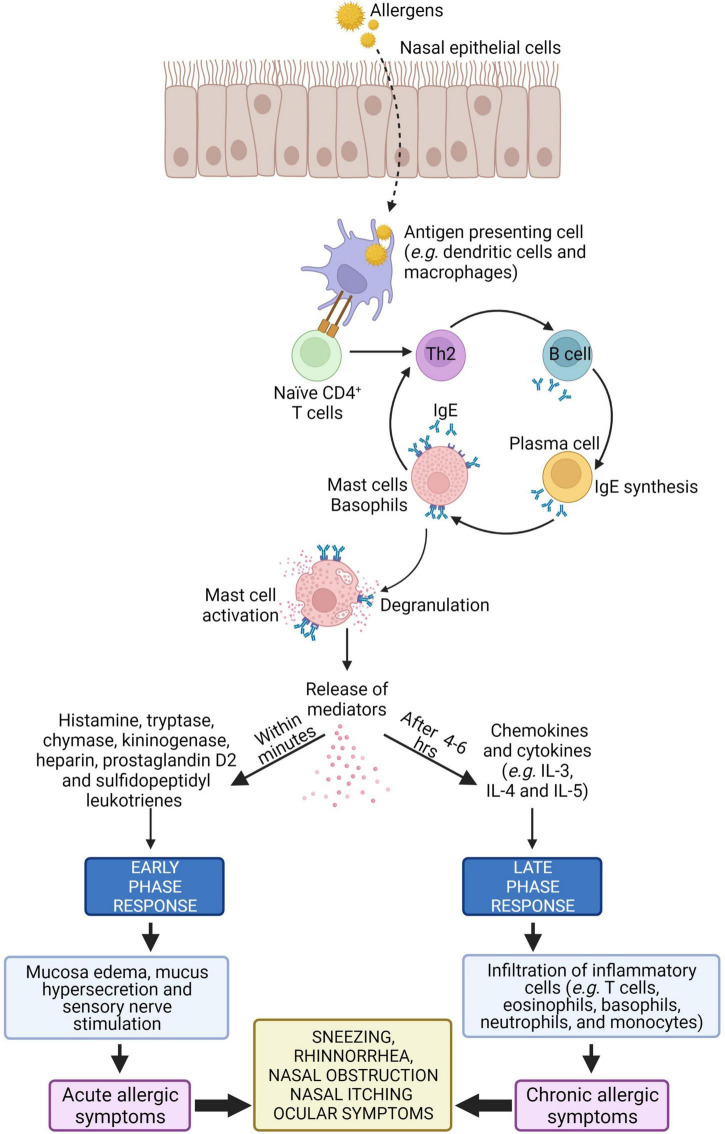
Schematic presentation of pathophysiology of AR (12, 59, 64, 66). See texts for details.

After 4–6 h of allergens exposure, the late phase of allergic response is initiated. In this phase, nasal mucosal inflammation occurs with the influx and activation of a variety of inflammatory cells such as T cells, eosinophils, basophils, neutrophils, and monocytes into the nasal mucosa (64). Recruitment of these inflammatory cells is triggered by cytokines such as IL-4 and IL-5. These cytokines upregulate the expression of adhesion molecules such as vascular cell adhesion molecule 1 (VCAM-1) on endothelial cells which facilitate inflammatory cellular influx (65). The activation of structural cells in the nasal mucosa, such as epithelial cells and fibroblasts, can promote the release of additional chemokines (e.g., eotaxin, RANTES, and TARC) that facilitate cellular influx from the peripheral blood (66). The schematic representation of pathophysiology of AR is illustrated in Figure 2.

### Allergens in Allergic Rhinitis

Allergens are typically proteins with molecular weight ranging from 10 to 40 kDa that induce type I hypersensitivity by reacting with specific IgE antibodies (28, 67). Common types of allergens include food allergens (e.g., shrimp, soybean, crab, clam, wheat, peanut, yolk egg, and cow’s milk), pet allergens (e.g., cat and dog dander), and HDMs (68, 69).

Major indoor allergens (e.g., HDMs, cockroaches, cat, and dog dander) have been consistently demonstrated to be the strongest risk factor for AR (70). Lifestyle heavily impacts the diversity and composition of the airway and gut microbiotas. The hygiene hypothesis proposes the importance of symbiotic relationship with relevant microorganisms in boosting immune system maturation and shifting the immune system toward a more tolerogenic state, and it thus suggests to be the fundamental cause for allergy onset (71).

**FIGURE 3:**
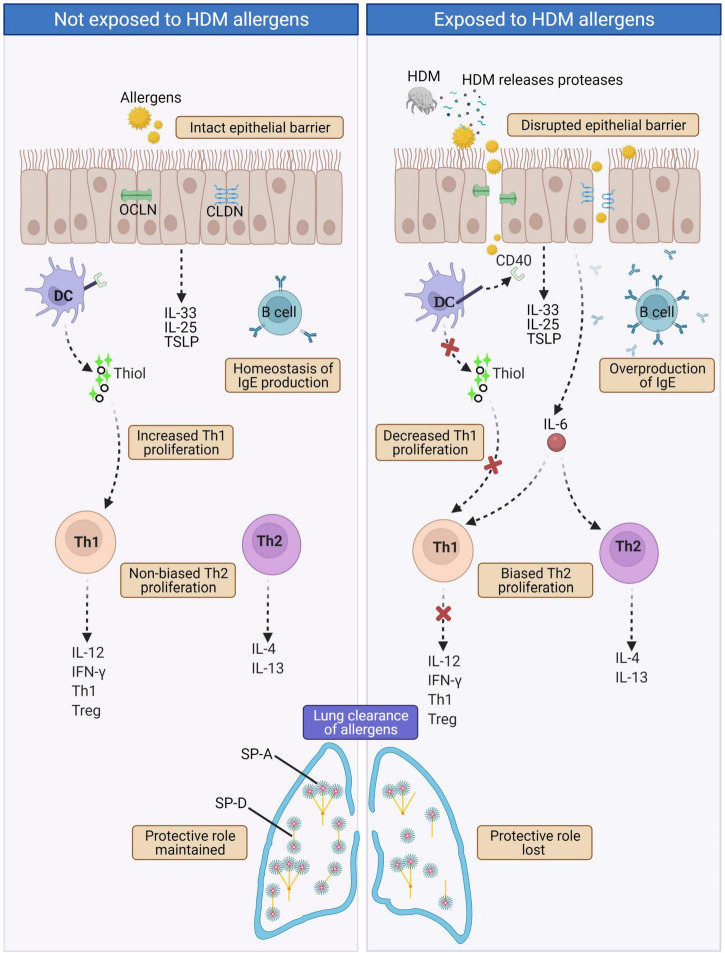
In normal physiological state (left panel), intact epithelial barrier prevents allergens infiltration and hence homeostasis of immune components and functions are maintained. In AR such as HDM-sensitized AR (right panel), proteases released by HDMs disrupt tight junctions leading to disrupted epithelial barrier that allows infiltration of allergens. This triggers a cascade of IgE overproduction by B cells, cleaved CD40 on the surface of DCs disrupts the production of thiols by DCs causing decreased Th1 proliferation and collectively with increased IL-6 secretion leads to biased Th2 proliferation. Th2 cells produce the hallmark AR cytokines IL-4 and IL-13. HDM proteases also cleave the pulmonary surfactants SP-A and SP-D, causing decreased lung clearance of allergens. CLDN, Claudin; DC, Dendritic cell; HDM, House dust mite; IL-4, Interleukin 4; IL-12, Interleukin 12; IL-13, Interleukin 13; IL-25, Interleukin 25; IL-33; Interleukin 33; IFNγ, Interferon gamma; OCLN, Occludin; SP-A, Surface protein A; SP-D, Surface protein D; Th1, T helper type 1; Th2, T helper type 2; Treg, Regulatory T cell; TSLP, Thymic stromal lymphopoietin.

In terms of HDM, one of the most common causes of AR, the protease activity of HDM leads to excessive IgE production. In normal physiological state, the level of IgE production by B cells is controlled by a negative feedback mechanism that involves IgE binding to CD23 (i.e., the low-affinity receptor for IgE FceRII). Upon binding of IgE/allergen-complexes to CD23, IgE production by B cell is then downregulated (72). However in HDM-sensitized AR patients, Der p 1 (a HDM cysteine proteinase allergen) disrupts this IgE-feedback mechanism via selective cleavage of CD23, causing overproduction of IgE by B cells (73). Lastly, in allergic diseases, pulmonary surfactants [i.e., surface protein (SP)-A and SP-D)] are vital in the clearance of allergens (72). They bind the allergens and lessen allergic sensitization by allergen removal or interference with IgE-binding (74, 75). Der p 1 has been shown to cleave SP-A and SP-D, leading to decreased lung clearance of allergens (76). A summary of the effects resulting from proteolytic activity of HDM is presented in Figure 3.

Apart from HDMs, previous reports have demonstrated that pollen allergens also impair tight junction (TJ) barrier function of epithelial cells. Pollens conferred proteolytic activities by degrading the TJ occludin in monolayers of Calu-3 cells (lung cancer cells of epithelial origin), resulted in increased paracellular permeability of the cells (77). Reduced expression of another TJ protein, CLDN1, enhanced Calu-3 cell transepithelial permeability. This has also been reported for pollen allergens widespread in the Mediterranean area (e.g., olive tree, orchard grass, Italian cypress, and Scots pine) where their exposure increased Calu-3 transepithelial permeability by disrupting TJ proteins (78). Likewise in Asia, 32.4% of self-reported pollen-induced AR was reported in the northern grassland region of China, a region with frequent occurrence of seasonal pollen (79).

### T Helper 2 Responses in Allergic Rhinitis

T Helper 2 (Th2) cells activate type 2 responses by stimulating B cells to proliferate and differentiate into plasma cells through the production of Th2 cytokines including IL-4, IL-5, IL-6, and IL-13 (80). Th2 cells are major contributors of IgE-producing B cells (81, 82), and Th2 cells play a predominant role in AR pathogenesis. Together with eosinophils and basophils, Th2 cells infiltrate the nasal mucosa tissue, resulting in late phase allergic response (83). IL-4 is a key cytokine in promoting Th2 differentiation from naïve CD4^+^ T cells (84). The mechanism is dependent on the activation of signal transducer and activator of transcription 6 (STAT6) signaling through IL-4 receptor complex.

Th2 cytokines not only enhance inflammatory cell activation but also may deregulate epithelial cell barrier integrity in allergic disease (e.g., AR, eosinophilic esophagitis, asthma, and chronic rhinosinusitis) (4, 5, 84–87). The cytokines may also be released within the sinonasal microenvironment including sinonasal epithelial cells, causing increased epithelial cell permeability (88, 89). This is thought to be due to regulation of transmembrane transcription involved in TJ remodeling where the “tight” barrier properties of TJ proteins are switched to “leaky” properties (89, 90). Th2 cytokines also hinder the epithelial barrier from resealing which may maintain the inflammation and exposure to inflammatory antigens (88).

## Treatment of Allergic Rhinitis

### Symptomatic Therapies in Allergic Rhinitis

Pharmaceutical management of AR rests on symptomatic treatments with antihistamines, nasal or oral glucocorticoids, nasal decongestants and leukotriene receptor antagonists that act as symptoms reliever in AR. Antihistamines is the most utilized first line medication to treat mild AR, however, first generation of antihistamines (e.g., diphenhydramine and hydroxyzine) are no longer recommended due to various adverse side effects impacting the central nervous system, anticholinergic side effects and cardiac toxicity (91–93). Newer generation of antihistamines (e.g., cetirizine, loratadine, desloratadine, fexofenadine, rupatadine, and bilastine) should be chosen as they demonstrate enhanced efficacy and safety profile (94). A newer type of intranasal antihistamine (e.g., olopatadine, levocabastine, and azelastine) ensures improved drug delivery to nasal mucosa exposed to release mediators during allergic inflammation in AR (95).

Besides, intranasal corticosteroid that acts as first-line pharmacotherapy by suppressing immune cells infiltration in AR is effective for both mild and moderate-severe AR in both children and adults (96). Currently approved intranasal corticosteroid for children are mometasone furoate (≥3 years old), fluticasone propionate (≥4 years old), triamcinolone acetonide (≥4 years old) and ciclesonide (≥6 years old) (97). A meta-analysis study was conducted to determine which combination therapies resulted in improved symptoms in AR patients. The meta-analysis demonstrated that intranasal H1 antihistamines and intranasal corticosteroids combination therapies were better compared with oral H1 antihistamines plus intranasal corticosteroid combination therapies (98).

Next, leukotriene receptor antagonists (e.g., montelukast, zafirlukast, and pranlukast) block the activity of cysteinyl leukotrienes, an important potent allergic mediator that causes allergic inflammation and various allergic symptoms such as nasal congestion and mucus production (94, 99). A meta-analysis study showed higher efficacy of leukotriene receptor antagonist compared with H1 antihistamines during nighttime symptoms but not in daytime symptoms (100). Other meta-analysis studies demonstrated that combination therapy of leukotriene receptor antagonist plus H1 antihistamines conferred increased efficacy in reducing daytime symptoms (101, 102).

Treatment using nasal decongestants reduces nasal congestion symptoms through their agonistic action at α1 and α2-adrenergic receptors on endothelial cells of nasal mucosa, leading to reduced mucosa swelling (103). Available nasal sprays in stores are oxymetazoline (Afrin), phenylephrine (Neo-synephrine) and pseudoephedrine (Sudafed). Overuse of nasal decongestants can cause rhinitis medicamentosa (i.e., a condition of rebound congestion upon withdrawal of nasal decongestants) (104) and this condition can be treated by administering intranasal corticosteroid (105, 106).

### Immunomodulating Therapies in Allergic Rhinitis

Treatment of AR targeting immune modulation aims to modify the natural courses of AR rather than to cause a shift to an immunologically naive or unresponsive state. In this section, we focused on allergen immunotherapy (AIT) in AR. A fraction of AR patients do not respond toward treatment with conventional pharmacotherapy, thus disease-modifying therapeutic agents are adopted such as AIT. Administration of AIT can be delivered either through subcutaneous (SCIT) or sublingual (SLIT) route. AIT of HDM-SCIT can induce temporary increase in FcεRI expression on DCs, but not on basophils, demonstrating potential tolerogenic roles of IgE/FcεRI signaling in DCs in the setting of AIT (107).

Basophils are important mediators in initiating early phase responses in AR, thus targeting these biomarkers through AIT is promising. One year therapy with SLIT for Parietaria can reduce the threshold of basophil activation which highlights the importance of AIT in treating disease and halting the disease progression (108). High serum periostin levels were observed after HDM-SLIT, indicating that serum periostin appears to be a useful biomarker in AIT (109). Furthermore, multiple cytokines profiling was investigated in treated AR patients with SLIT where serum BAFF, IFNγ, IL10, and IL33 levels were strongly predictive of the efficacy of SLIT (110). These sets of biomarkers are closely associated with Th1 immune response and type 2 innate lymphoid cells downstream signaling pathway in AR.

Another independent study measuring SNOT-20 score after SLIT treatment in AR reported a reduction in AR symptoms and improvement in QOL of AR patients (111). AIT is proven to be a safe treatment for pediatrics AR. Adverse reactions related to SCIT in pediatric patients were reported to be infrequent, and grade 1 adverse reactions being the most frequently reported (112). In the recent large retrospective cohort study of AIT clinical trial REACT (real-world effectiveness in allergy immunotherapy) (113), it involved 46,024 AIT-treated subjects composed of AR patients with or without asthma vs. control subjects without AIT treatment. The AIT group exhibited greater reduction in AR and asthma prescriptions together with reduced severe asthma exacerbations. The REACT clinical trial demonstrated longer and sustained effectiveness of AIT in real-world settings.

## Conclusion and Future Directions

Increased prevalence of AR and that it is an incurable disease pose major unmet needs to alleviate these issues. AR comorbidities also confer increased health and socioeconomic burden on AR patients. Moreover, diagnosing AR is more challenging in patients with dual AR (DAR), a recently defined AR phenotype in which the DAR patients display perennial and seasonal allergies-related nasal symptoms, and only allergic to seasonal allergies. This indicates the importance of measuring inflammation at local allergic sites which might be different from peripheral inflammation in DAR patients (114–116). Thus, future studies warrant a better understanding of allergen-mediated cellular mechanisms at local sites of AR patients, in conjunction with further research on its pathogenic mechanisms such as breakdown of nasal epithelial barrier integrity that may lead to the discovery of novel therapeutic agents for the disease.

## Author Contributions

SMNH, HTT, NSMA, and KKW conceived the manuscript. SMNH and KKW designed the manuscript, performed literature search, wrote and revised the manuscript. SMNH prepared the figures and table. HTT, NSMA, and NMS revised the manuscript. All authors have read and approved the final manuscript.

## Conflict of Interest

The authors declare that the research was conducted in the absence of any commercial or financial relationships that could be construed as a potential conflict of interest.

## Publisher’s Note

All claims expressed in this article are solely those of the authors and do not necessarily represent those of their affiliated organizations, or those of the publisher, the editors and the reviewers. Any product that may be evaluated in this article, or claim that may be made by its manufacturer, is not guaranteed or endorsed by the publisher.
